# Remote assessment of disease and relapse in major depressive disorder (RADAR-MDD): a multi-centre prospective cohort study protocol

**DOI:** 10.1186/s12888-019-2049-z

**Published:** 2019-02-18

**Authors:** F. Matcham, C. Barattieri di San Pietro, V. Bulgari, G. de Girolamo, R. Dobson, H. Eriksson, A. A. Folarin, J. M. Haro, M. Kerz, F. Lamers, Q. Li, N. V. Manyakov, D. C. Mohr, I. Myin-Germeys, V. Narayan, Penninx BWJH, Y. Ranjan, Z. Rashid, A. Rintala, S. Siddi, S. K. Simblett, T. Wykes, M. Hotopf, on behalf of the RADAR-CNS consortium, Sonia DiFrancesco, Katie White, Alina Ivan, Ashley Polhemus, Jose Ferrao, Michiel Ringkjøbing-Elema, Francesco Nobilia, Wolfgang Viechtbauer, Sjaak Peelen, Zulqarnain Rashid, Janneke Boere, Nicholas Cummins, Nick Meyer

**Affiliations:** 1 King’s College London, Institute of Psychiatry, Psychology and Neuroscience, London, UK; 2 IRCCS Istituto Centro San Giovanni di Dio Fatebenefratelli, Brescia, Italy; 3 Univeristy of Milan-Bicocca, Milan, Italy; 4 H. Lundbeck A/S, Valby, Denmark; 5 Parc Sanitari Sant Joan de Déu, Fundació Sant Joan de Déu, CIBERSAM, Universitat de Barcelona, Barcelona, Spain; 6 Department of Psychiatry and Amsterdam Public Health Research Institute, VU University Medical Centre, Amsterdam, The Netherlands; 7 Janssen Research and Development, LLC, Titusville, NJ USA; 8 Janssen Research and Development, LLC, Beerse, Belgium; 9 Center for Behavioral Intervention Technologies, Department of Preventive Medicine, Northwestern University, Chicago, IL USA; 10 Department for Neurosciences, Center for Contextual Psychiatry, KU Leuven, Leuven, Belgium; 11 https://www.radar-cns.org/

**Keywords:** Major depressive disorder, Remote measurement technology, Passive sensing, M-health, Prospective study, Observational cohort, Outcome measurement

## Abstract

**Background:**

There is a growing body of literature highlighting the role that wearable and mobile remote measurement technology (RMT) can play in measuring symptoms of major depressive disorder (MDD). Outcomes assessment typically relies on self-report, which can be biased by dysfunctional perceptions and current symptom severity. Predictors of depressive relapse include disrupted sleep, reduced sociability, physical activity, changes in mood, prosody and cognitive function, which are all amenable to measurement via RMT. This study aims to: 1) determine the usability, feasibility and acceptability of RMT; 2) improve and refine clinical outcome measurement using RMT to identify current clinical state; 3) determine whether RMT can provide information predictive of depressive relapse and other critical outcomes.

**Methods:**

RADAR-MDD is a multi-site prospective cohort study, aiming to recruit 600 participants with a history of depressive disorder across three sites: London, Amsterdam and Barcelona. Participants will be asked to wear a wrist-worn activity tracker and download several apps onto their smartphones. These apps will be used to either collect data passively from existing smartphone sensors, or to deliver questionnaires, cognitive tasks, and speech assessments. The wearable device, smartphone sensors and questionnaires will collect data for up to 2-years about participants’ sleep, physical activity, stress, mood, sociability, speech patterns, and cognitive function. The primary outcome of interest is MDD relapse, defined via the Inventory of Depressive Symptomatology- Self-Report questionnaire (IDS-SR) and the World Health Organisation’s self-reported Composite International Diagnostic Interview (CIDI-SF).

**Discussion:**

This study aims to provide insight into the early predictors of major depressive relapse, measured unobtrusively via RMT. If found to be acceptable to patients and other key stakeholders and able to provide clinically useful information predictive of future deterioration, RMT has potential to change the way in which depression and other long-term conditions are measured and managed.

**Electronic supplementary material:**

The online version of this article (10.1186/s12888-019-2049-z) contains supplementary material, which is available to authorized users.

## Background

The last decade has seen an explosion in the capability of monitoring individuals via sensors in smartphones or wearable devices, and the range of parameters which can be measured by such technologies will continue to grow [1]. The development of remote measurement technologies (RMT), using these inbuilt sensors to unobtrusively measure human behaviour and physiology, combined with active measurement of daily experiences via smartphone apps, is an innovation which could be used to provide real-time information about patients’ current clinical state, as well as information potentially predictive of future deterioration [1].

Major Depressive Disorder (MDD) is associated with a wide range of negative outcomes including: premature mortality [2]; reduced quality-of-life [3]; loss of occupational function [4]; and is often experienced alongside physical comorbidity [5] and approximately 55% will go on to develop chronic depression, characterised by periods of recovery and relapse [6, 7]. Developing interventions which can disrupt the pattern of remission and relapse is of crucial importance to maintain sustained periods of recovery [8]. Predictors of depressive relapse include disrupted sleep [9], reduced sociability [10], changes in mood [11], prosody [12] and cognition [13], and there is evidence highlighting the impact psychological and pharmacological interventions can have on reducing risk of relapse [14, 15]. Identifying the optimal time to deliver a preventative intervention, when these symptoms may be sub-threshold, context dependent, or too subtle for the individual to recognise, is challenging. Symptom recall in patients with depression may also not be accurate, potentially biased by current symptom severity and environmental stressors [16, 17]. This retrospective recall cannot capture symptom variability and context reactivity which would allow interventions to be administered at a crucial point in the relapse signature.

RMT may provide a solution to these problems by giving richer, objective characterisation of behaviour, cognition, speech and physiology. Data can be collected with excellent temporal resolution, including an ability to assess diurnal variation which may be indicative of early decline in mood [18]. Passive RMT (pRMT) uses sensors in activity monitors and smartphones to gather data with no input from the wearer [19], including Global Positioning System (GPS), accelerometer, gyroscope, communication logs, ambient noise and light levels and screen interactions. Many of these sensors have been used to identify changes in sleep, communication and activity patterns associated with depressive episodes [20, 21]. Prosodic speech characteristics, such as speaking rate, pitch, pause duration and energy, collected via smartphone microphones, have been used to detect depression with a prediction accuracy of 81.3% [22]. Active RMT (aRMT) uses smartphone apps to deliver validated questionnaires, cognitive games, speech tasks or electronic diaries using the experience sampling method (ESM) to provide fine-grained understanding of mood changes and stressors in the context of daily life [23]. aRMT has been used to measure affect, cognitions, thoughts, mood and behaviours in real-time, with evidence highlighting the increased validity of this methodology in comparison to traditional retrospective reports [24]. aRMT assessments of positive and negative affect have also been found to be reliably indicative of mood state [25, 26] and have been associated with MDD symptoms [27].

Despite this growing body of research evidence, there has been limited assessment of the utility of multi-parametric RMT in clinical populations [28, 29], combining multiple sensors to detect signatures potentially useful for predicting outcomes in MDD. This protocol describes the clinical study designed to test this utility of RMT.

### Patient involvement in the RADAR-MDD study protocol

Systematic reviews have identified the barriers and facilitators of RMT uptake [30]; service-user focus groups have identified the outcomes prioritised by service-users, identified appropriate methods of measuring these outcomes, and investigated the use of technology to measure and manage symptoms [31]; surveys and user experience testing have informed the selection of devices and development of all user-facing systems to ensure optimal usability and functionality. Extensive device-selection procedures combined all acquired knowledge from literature reviews, focus groups and scoping exercises to provide a short-list of suitable activity monitors. Participant-facing documents (such as information sheets and consent forms) were developed in collaboration with service-user research groups to ensure materials are understandable and describe the technology and protocol in clear language.

### Study objectives

RADAR-MDD is a clinical study aiming to assess the utility of multi-parametric RMT in clinical populations with MDD.

Specifically, RADAR-MDD aims to:

1. Determine the usability, feasibility and acceptability of, and adherence to, RMT to provide real-time objective multidimensional indications of clinical state in individuals with MDD.
2. Improve and refine clinical outcome measurement using RMT as a means of identifying current clinical state.
3. Determine whether multi-parametric RMT collected in populations with recurrent MDD can provide information predictive of depressive relapse and other critical outcomes including anxiety, quality-of-life, and MDD remission.

## Methods

### Study design

RADAR-MDD is a multi-centre prospective observational cohort study in which 600 individuals with a recent history of MDD will be asked to download several RMT apps and use an activity tracker for up to 2 years of follow-up. The study involves no randomisation or intervention, and uses commercially available activity trackers and smartphone sensors.

### Study population

An estimated 100 relapses are required in order to provide sufficient power on which to test the predictive utility of RMT, if 10 variables were entered into the predictive model [32]. With an approximated annual relapse rate of 33% (a lower estimate than that provided in the Star*D trial; [33]), 300 participants would be required, followed-up for one year. Although there is evidence to suggest promising uptake to RMT protocols, the attrition rate in this length of follow-up is unknown, there are likely to be missing data, data are inherently “noisy”, and the relapse rate in our population may be lower than anticipated. We therefore aim to recruit a total of 600 individuals with a history of MDD across three research sites.

The eligibility criteria for inclusion in this study are summarised in Table 1. To recruit people at risk of relapse during the follow-up period, people with a recent history of MDD, with indication of a recurring trajectory will be approached to participate. No limitations have been applied regarding any treatment they may be receiving, although medication use and other psychological interventions will be monitored throughout the course of follow-up. Additionally, participants will be eligible to participate regardless of whether they are currently experiencing depression symptoms or not.

**Table 1:** Eligibility criteria for participation in RADAR-MDD

Inclusion criteria	Exclusion criteria
Meet DSM-5 diagnostic criteria for diagnosis of non-psychotic MDD within the past 2 years. Recurrent MDD (a lifetime history of at least 2 episodes of depression; (LIDAS) [34]). Willing and able to complete self-reported assessments via smartphone. Able to give informed consent for participation. Fluent in English, Spanish, Catalan or Dutch language Existing ownership of Android smartphone or willingness to use an Android smartphone as their only smartphone. Aged 18 or over	Lifetime history of bipolar disorder, schizophrenia, MDD with psychotic features, schizoaffective disorders. Dementia History of moderate to severe drug or alcohol dependence within the last 6 months History of major medical disease which might impact upon the patient’s ability to participate in normal daily activities for more than 2 weeks (e.g. due to likely hospitalisations or other periods of indisposition). Pregnancy

### Study procedures

**Fig. 1:**
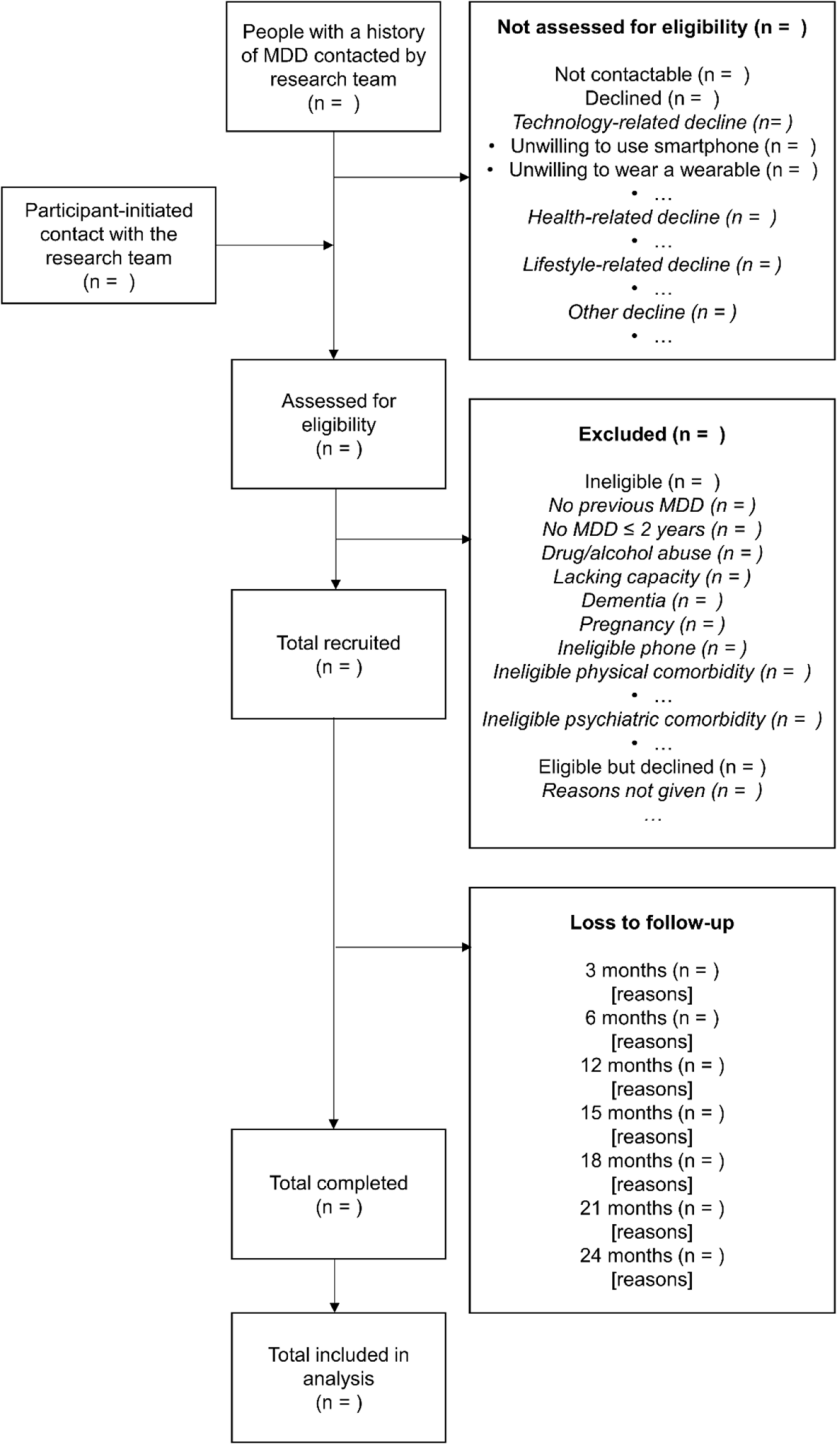
Participant flowchart

Participants will be recruited over eighteen months from three international clinical cohorts or research infrastructures held at King’s College London (UK); the Netherlands Study of Depression and Anxiety and other available patient groups at Vrije Universiteit Medisch Centrum (VUmc; Amsterdam, The Netherlands); and Centro de Investigacion Biomedican en Red (CIBER; Barcelona, Spain). The schedule of observations is available in Table 2. The flow of participants through the study, including reasons for not participating in the study will be standardised across sites and documented in a flowchart (Fig. 1).

**Table 2:** Schedule of events for RADAR-MDD

Month	-1	0	1	2	3	4	5	6	7	8	9	10	11	12	…	23	24
Visit	0	1	n/a	n/a	n/a	n/a	n/a	n/a	n/a	n/a	n/a	n/a	n/a	n/a	n/a	n/a	2
Study explanation (T)	X																
Informed consent (F2F)		X															
Introductory training (F2F)		X															
Baseline assessments																	
Socio-demographics (R/F2F)		X															
Social Environment (R/F2F)		X															
Medical History (R/F2F)		X															
Depression History (R/F2F)		X															
Process evaluation																	
Qualitative interview (F2F/T)					X									X			X
TAM (R)					X									X			X
PSSUQ (R)					X									X			X
Remote Data Collection																	
Wearable sensors			Continuous months 1–24														
Smartphone sensors (pRMT)			Continuous months 1–24														
ESM assessment (aRMT)*		X	X	X	X	X	X	X	X	X	XX	X	X	X	X	X	
Speech (aRMT)		XX	XX	XX	XX	XX	XX	XX	XX	XX	XX	XX	XX	XX	XX	XX	XX
Mood, PHQ8 (aRMT)		XX	XX	XX	XX	XX	XX	XX	XX	XX	XX	XX	XX	XX	XX	XX	XX
Self-esteem RSES (aRMT)		XX	XX	XX	XX	XX	XX	XX	XX	XX	XX	XX	XX	XX	XX	XX	XX
Cognition (THINC-IT) *		X	X	X	X	X	X	X	X	X	XX	X	X	X	X	X	
Outcome assessment																	
Relapse IDS-SR/CIDI-SF (R)		X			X			X			X			X			X
Anxiety GAD7 (R)		X			X			X			X			X			X
Quality-of-life WSAS (R)		X			X			X			X			X			X
BIPQ (R)		X			X			X			X			X			X
Remission IDS-SD/CIDI-SF (R)		X			X			X			X			X			X
CSRI (R)		X			X			X			X			X			X
LTE-SR (R)		X			X			X			X			X			X
Treatment information (R)		X			X			X			X			X			X
Alcohol use AUDIT (R)		X			X			X			X			X			X
Clinical Interview (F2F/T)		(X)			(X)			(X)			(X)			(X)			(X)

T: Telephone. F2F: face-to-face. R: REDCap web-based platform. pRMT: passive remote measurement app. aRMT: active remote measurement app. (X): clinical interview only conducted in people identified as MDD relapse cases by IDS-SR/CIDI-SF primary outcome measurement. X delivered once per month. XX delivered twice per month (every 2-weeks). *ESM and cognition tests are conducted once every 6 weeks

Recruitment procedures will vary slightly across sites. Potentially eligible individuals will be identified either through existing research cohorts (in London and The Netherlands) who have given consent to be contacted for research purposes, or through mental health services (in London and Barcelona). These individuals will be contacted by a member of the on-site research teams and sent the study information sheet and consent from. Those who provide verbal consent to participate will have a more detailed conversation with the research teams, during which they will have an opportunity to ask questions about the study, have their eligibility confirmed, and arrange a suitable time for enrolment.

Enrolment will either happen at the research centre, or at the participant’s home depending on the site they have been recruited from. Participants will receive a £15/€20 for enrolling in the study, and £5/€10 for every three months of continued participation. Participants who take part in additional qualitative interviews at 3-month or 1-year follow-up will receive £10/€10 per interview. Written consent will be obtained, during the enrolment session, which will include collection of sociodemographic, social environment, medical history and technology use questionnaires, and lifetime depression history measured through the Lifetime Depression Assessment – Self-Report (LIDAS [34];), alongside baseline data collection of all outcome measures via Research Electronic Data Capture (REDCap) software [35].

### Remote data collection

RADAR-MDD is the clinical study in depression being undertaken as part of a larger body of work managed by the Remote Assessment of Disease and Relapse – Central Nervous System (RADAR-CNS; [36]) international research consortium. This includes similar clinical studies conducted in epilepsy and multiple sclerosis, as well as teams dedicated to patient and public involvement, integration into care pathways, regulation. Software engineers, data scientists and software developers have developed an open-source platform to support data collection via wearable devices. More detailed technical information about this open-source platform and the sensors available for use is provided elsewhere [37]. Below is a summary of the methods of remote data collection used in the RADAR-MDD study.

#### Passive RMT (pRMT) app

All modern smartphones comprise the sensors required for measurement in RADAR-MDD, however our study will only incorporate Android smartphones. For participants who do not use a smartphone, we will offer to provide a suitable smartphone for them. Participants who are using a non-Android smartphone will not be recruited unless they explicitly state that they are willing to switch to an Android smartphone as their only phone for the duration of follow-up, and in these cases an appropriate phone will be provided. Each participant will be asked to install the purpose-built pRMT application. This will run in the background, requiring minimal input from participants, collecting data on ambient noise, ambient light, GPS location, keystokes (at the London site only), Bluetooth connectivity, length and duration of calls, number of text messages and emails and weather conditions, in addition to battery life. GPS location data will be obfuscated; that is, providing relative location data, not absolute coordinates, preventing identification of an individual’s home address or precise geographical location.

#### Active RMT (aRMT) app

Following extensive focus group work and user testing [31], an additional app has been created to administer the aRMT aspects of the study protocol. This includes validated questionnaires to measure depressive symptoms and self-esteem. Variability of depressive symptoms will be measured via the 8-item Patient Health Questionnaire (PHQ8; [38]) every 2 weeks throughout the course of follow-up. Variation in self-esteem will be measured using the Rosenberg Self-Esteem Scale; RSES [39]. The RSES is a widely-used 10-item self-reported questionnaire used to quantify self-esteem along a continuum and will be administered alongside the PHQ8 every 2 weeks.

As with the PHQ8 and RSES, every 2- weeks participants will be asked to complete a speech task. This requires participants to say aloud, in a quiet area, some excerpts from “The North Wind and the Sun”, which are shown to be phonetically balanced across all three languages [40]. These will be offered on a random schedule to prevent rehearsal and fluency and preserve prosodic features. In addition to this, participants will be asked to respond to the following question: “Can you describe something you are looking forward to this week?”

The aRMT app will also deliver an ESM schedule (see online Additional file 1), which has been designed to collect brief, in-the-moment assessments relating to several of our domains of interest: mood, stress, sociability, activity and sleep. Participants will receive a series of questions intended to reflect their current state (such as “right now, I feel content”), with 7-point Likert scale answer options (0 = Not at all, 7 = Very much). The ESM schedule consists of approximately 44 items, taking up to 3-min to complete, delivered 9 random times per day within 90-min blocks starting from 08.30 and ending at 22.00 for 6 consecutive days every 6 weeks.

Cognitive function will also be measured using the THINC-IT® app, which has been specifically designed and validated to screen for cognitive dysfunction in depression [41], and includes assessments of elements of executive function such as memory, concentration, and attention.

#### Wearable sensors

The wearable device planned for use in RADAR-MDD is the FitBit Charge 2. Participants will be asked to wear this device for the duration of follow-up, providing ongoing data collection of accelerometery and heart rate, which provide information about movement, daytime activity, sedentary activity, physical exercise, step count, sleep efficiency, sleep latency, wake episodes, sleep fragmentation and post-midnight sleep start. The FitBit Charge 2 was selected due to its commercial availability, its competitive pricing, and the range of measurements available. Furthermore, it met several usability requirements highlighted by our focus groups and patient advisory boards, including: additional utility of a watch-face; comfort and durability; provided by a well-known brand (therefore reducing any potential stigma or identification as “research participant”); an acceptable battery life (3–5 days, requiring approximately 30 min to charge fully), and an excellent app interface. In research environments, the FitBit has also been found to be acceptable for use by depressed samples across a relatively long period of follow-up [42], and has recently demonstrated good ability to accurately identify sleep-wake state and sleep stage cycles [43].

### Outcome measurements

Measurements will be taken at baseline, then every three months for the duration of follow-up (up to 2 years). All outcomes will be collected via Research Electronic Data Capture (REDCap) software [35], which sends automatic survey invitations and reminders to participants for the duration of the study.

#### Primary outcome

##### Major depressive disorder relapse

The presence of MDD during follow-up will be defined as meeting criteria for MDD according to the Wold Health Organisation’s Composite International Diagnostic Interview – Short Form (CIDI-SF; [44]). The CIDI-SF has been used extensively on web-based platforms, and has shown excellent sensitivity and specificity to identify current MDD state [44]. Additionally, a score of > 25 on the the Inventory of Depressive Symptomatology – Self-Reported (IDS-SR; [45]) will be required to establish that the severity of the depressive episode is at least moderate. The IDS-SR is a 30-item questionnaire, widely used in depression trials and providing detailed information on depressive symptoms, including all 9 Diagnostic and Statistical Manual (DSM) domains, symptoms commonly associated with depression (such as irritability or pain), melancholic symptoms and atypical symptoms. Scores on each of the 30 items are summed to create a total score ranging from 0 to 84, with the following thresholds: 0–13 (no symptoms); 14–25 (mild symptoms); 26–38 (moderate symptoms); 39–48 (severe symptoms); 49–84 (very severe symptoms) [45]. In the case of IDS-SR/CIDI-SF identified relapse, a confirmatory diagnostic interview will be conducted, to additionally confirm of relapse onset.

#### Secondary outcomes

*Anxiety* will be measured via the 7-item Generalised Anxiety Disorder questionnaire; GAD7 [46], which provides a dimensional score of anxiety symptoms which are frequently comorbid with depression. *Quality-of-life/disability* will be measured via the Work and Social Adjustment Scale; WSAS [47], which is a 5-item assessment of perceived social and work-related functional impairment used widely across a range of mental and physical disorders. *Illness perceptions* will be assessed using the Brief Illness Perceptions Questionnaire; BIPQ [48]. The BIPQ provides an insight into the participant’s views about their underlying condition – including a measure of how well they see themselves coping with its symptoms and treatments. *Alcohol use* will be measured using the self-reported Alcohol Use Disorders Identification Test (AUDIT), which has been found to be a reliable and valid method of identifying at-risk and alcohol-dependent drinkers [49]. *MDD remission*, the confirmation of continued remission status, or new remission status in participants who had previously relapsed, will be established using the IDS-SR followed by CIDI-SF. Participants scoring < 14 on the IDS-SR and who do not meet CIDI-SF criteria for MDD will be considered in a state of remission.

#### Other measurements

We will also seek to identify major changes in participants’ circumstances which may affect outcomes as well as use of RMT (such as the death of a relative or major illness), so every 3 months we are additionally collecting information about: treatments for depression, including changes in antidepressants, psychological therapies and any other action the individual has taken to alleviate depressive symptoms, and adherence to medication. The modified Client Service Receipt Inventory (CSRI [50]) is a well-validated and widely used measure of health economic data allowing us to model costs in future analyses. Change in life circumstances will be assessed using the List of Threatening Experiences – Questionnaire; LTE-Q [51].

#### Process evaluation

To ensure a system which meets participant and researcher needs, there will be continued monitoring of the acceptability and usability of the RADAR-CNS platform, including the app, assessment scheme and outcome collection platform. This will involve conducting qualitative interviews with a random selection of 15 participants enrolled within the first 3 months and thorough assessment of platform usability via self-reported questionnaires (the Post-Study System Usability Questionnaire (PSSUQ [52]) and the Technology Assessment Model Fast Form (TAM-FF; [53]). This process will be replicated in a further 15 randomly selected participants at the point of drop-out or at 1-year post-enrolment. At study endpoint, post-participation qualitative interviews will be conducted in a random selection of participants who participated until study end-point, and those who dropped out throughout the course of follow-up. Data obtained throughout the course of process evaluation will be used, if necessary, to make iterative developments to the platform software, to optimise usability of the system.

#### Follow-up procedure and data monitoring

Participants will be encouraged to live their lives as normal, responding to in-app notifications when required and completing outcomes assessments via REDCap every 3 months for the duration of follow-up. The research team will be able to periodically review incoming data using the RADAR-CNS management portal and may contact participants if there is a loss of data stream from a device, to determine whether there are issues relating to functionality or user-experience. These contacts will be recorded as evidence of feasibility and acceptability outcomes. In addition to the telephone call provided after the introductory training session, participants will receive a further call after 1-month to address any further concerns or questions, and brief follow-up telephone calls will then be offered every 3 months to maintain engagement with participants throughout the course of the study and to remind participants of any upcoming outcome assessments.

In the case of suspected depressive relapse, participants will also be contacted by research teams to perform clinical interview and risk assessment. In the case of a depressive relapse or for participants who express significant suicidal ideas, we will advise the participant to contact their treating physician. If the participant is not willing to do so, the study team may, if sufficiently concerned, make direct contact with the treating physician without participant consent. Note that participants will, at recruitment, be forewarned and be asked to consent to this procedure.

### Study debrief

At the end of the follow-up period, participants will receive a maximum 60 min “debrief” session, which will serve to collect end-point acceptability and usability outcomes, and include a qualitative interview investigating their experiences of participating for randomly selected participants. This will be done in person if possible, or over the phone if more convenient for the participant. Participants will also have an opportunity to view and discuss their own data.

### Adverse events and study withdrawal

It is not expected that participation in RADAR-MDD will increase the risk of relapse, nor of there being other significant risks of harm to participants. There may be several reasons for withdrawal from the study:

1. Participant choosing to no longer participate; participants will be informed both in writing and verbally that participation is voluntary, that non-participation will not influence their medical care, and that they are free to withdraw from the study at any point without providing reasons.
2. The research team may withdraw the participant in the event of inter-current illness, adverse event (AE), protocol violation, administrative or other reasons.
3. The participant loses capacity for continued participation.

In the case of participant self-withdrawal, all attempts will be made to follow-up with the participant to establish cause of withdrawal, and to collect qualitative data regarding experience of participation. Depressive relapse itself will not be cause for automatic withdrawal; unless they choose to stop, participants who are identified as experiencing a relapse will continue to participate until study end-point. All data, including those from withdrawn participants (unless they request for their data to be deleted), will be included in the final analysis. If a participant withdraws from the study prematurely without providing evaluable data, a replacement participant will be sought if resources allow.

### Statistical and analysis plan

Descriptive statistics for demographics, attrition rate, and number of participants using the remote assessment measurements without loss or damage and providing adequate data for the duration of the study period will be estimated. Using classification/regression approaches, we will investigate whether any demographics and/or other numerical information obtained during baseline of the study might serve as a predictor for subjects drop out/percentage of adequate data. Using mixed-effect models with participants as random factor, we will estimate whether relationships between the amount of usable data during the weeks prior to the outcome assessment and obtained scale’s measurements exist.

Aggregated features obtained from biosensors, smartphones, cognitive tests and from ESM, as well as their changes from baseline or previous time points (delta features) will be used for statistical analysis. Aggregation will be done for the period of 2 weeks prior to participant’s evaluation with PHQ8 or longer time interval preceding 3-mothly outcome scale assessment, since relapse identification is done retrospectively. First, univariate approach, where each variable/feature will be modelled with use of mixed-effect models, with depressive relapse as fixed and participant as random effect, to find out which variables are influenced during the relapse. Demographics and other baseline characteristics will be added to the model, when necessary, with correction for multiple comparisons.

Secondly, we will construct predictive models, using aggregated data obtained prior to assessment of relapses and their changes from previous time points to predict binary (relapsed or not) or continuous (when scale score is treated as continuous variable) outcomes. Under assumption that data are missing at random, multiple imputation will be applied for covariates, where missing-ness is not drastically high. Multivariate prediction model will be constructed and variable selection, based on, for example, LASSO L1-regularization for linear models, or different feature selection algorithms will be applied. Models’ performance will be characterized through the cross-validation, putting stress on sensitivity and specificity of relapse prediction model.

## Discussion

There is substantial evidence to suggest that changes in physical activity, sleep, stress, prosodic features, cognitive function, mood and sociability are associated with an increased risk of relapse [9–13]. RMT provides a new and unobtrusive way of measuring these variables, using smartphones and wearable technology to collect data reflective of participants’ daily lives and clinical symptoms. RADAR-MDD is the largest study to investigate multimodal RMT in a clinical cohort. If RADAR-MDD demonstrates that RMT is feasible to use in large populations, acceptable for participants, and provides clinically useful data, it may represent that start of a paradigm shift in how clinical data are collected and respective change in how health services are delivered [54]. The RADAR-CNS platform is a device-agnostic system and could be applied to any diagnosis or incorporated into any study design, potentially transforming data collection for clinical and research purposes.

However, to ensure translation into health services, extensive work is required to identify the RMT biosignatures most useful to assist in early detection of deterioration, and to understand the potential barriers and facilitators of large-scale uptake. There are anticipated challenges to this novel method of data collection. Firstly, change in health status may be a key barrier to long-term engagement with this study [30]; an exacerbation of depressive symptoms may influence ongoing participation rate throughout the duration of follow-up, and participant drop-out may represent a depressive relapse. However, there is also some evidence to suggest that people experiencing more severe symptoms may participate more fully, as they may be more motivated to improve their understanding of their condition [55]. Secondly, whilst this is a non-interventional study, encouraging individuals to monitor their own health and providing feedback on activity and sleep (via the FitBit app) may prompt people to change their behaviours or seek help sooner, reducing risk of relapse. There is also evidence to suggest that encouraging health monitoring may increase health anxiety, which in turn may contribute to worsening depressive symptoms [31].

There are clear challenges to overcome, and RADAR-MDD hopes to identify as many of them as possible through qualitative interviews and intensive follow-up procedures and provide some insight into how to overcome these barriers for future implementation of RMT procedures. There are also important future benefits of this method of data collection. The data are inherently personal, and providing personalised feedback may increase individuals’ health awareness and facilitate self-management behaviours to improve long-term health outcomes [56]. Furthermore, RMT may provide a solution to traditional reliance on infrequently measured subjective outcomes in clinical trials, which can lead to imprecision, inefficiency, and the need for larger sample sizes.

RADAR-MDD is an essential step towards improving our understanding of the feasibility and acceptability of large-scale RMT data collection and determining the clinical utility and predictive ability of RMT to predict relapse in major depressive disorder.

## Additional file

Additional file 1: Esperience Sampling Methodology (ESM) assessment scheme. (DOCX 19 kb)

## Abbreviations

AE: Adverse event
aRMT: Active remote measurement technology
AUDIT: Alcohol use disorders identification test
BIPQ: Brief Illness perceptions questionnaire
CIBER: Centro de investigacion biomedican en red
CIDI-SF: Composite international diagnostic interview - self Report
CSRI: Client service receipt inventory
DSM: Diagnostic and statistical manual
ESM: Experience sampling method
GAD7: 7-item generalised anxiety disorder questionnaire
GPS: Global positioning system
IDS-SR: Inventory of depressive symptomatology- self-report questionnaire
LIDAS: Lifetime depression assessment – self-report
LTE-Q: List of threatening experiences – questionnaire
MDD: Major depressive disorder
PHQ8: 8-item patient health questionnaire
pRMT: passive remote measurement technology
PSSUQ: Post-Study system usability questionnaire
RADAR-CNS: Remote assessment of disease and relapse - central nervous system
RADAR-MDD: Remote assessment of disease and relapse - major depressive disorder
REDCap: Research electronic data capture
RMT: Remote measurement technology
RSES: Rosenberg self-esteem scale
TAM-FF: Technology assessment model fast form
VUmc: Vrije Universiteit Medisch Centrum
WSAS: Work and social adjustment scale
