# Supplementary material for: Remote assessment of disease and relapse in major depressive disorder (RADAR-MDD): a multi-centre prospective cohort study protocol
Source: BMC Psychiatry. 2019 Feb 18;19:72. doi: 10.1186/s12888-019-2049-z (PMC6379954; doi:10.1186/s12888-019-2049-z)
Supplement: Supplementary file 1 — Esperience Sampling Methodology (ESM) assessment scheme. (DOCX 19 kb) [file 12888_2019_2049_MOESM1_ESM.docx]

# Additional file 1

| Diary questionnaire | Scale option |
| --- | --- |
| I slept well (once per day) | Not at all 0 1 2 3 4 5 6 7 Very much |
| Right now, I feel cheerful | Not at all 0 1 2 3 4 5 6 7 Very much |
| Right now, I feel down | Not at all 0 1 2 3 4 5 6 7 Very much |
| Right now, I feel anxious | Not at all 0 1 2 3 4 5 6 7 Very much |
| Right now, I feel relaxed | Not at all 0 1 2 3 4 5 6 7 Very much |
| Right now, I feel irritated | Not at all 0 1 2 3 4 5 6 7 Very much |
| Right now, I feel stressed | Not at all 0 1 2 3 4 5 6 7 Very much |
| Right now, I feel content | Not at all 0 1 2 3 4 5 6 7 Very much |
| Right now, I feel insecure | Not at all 0 1 2 3 4 5 6 7 Very much |
| Right now, I feel hopeful | Not at all 0 1 2 3 4 5 6 7 Very much |
| Right now, I feel lonely | Not at all 0 1 2 3 4 5 6 7 Very much |
| I am satisfied with myself | Not at all 0 1 2 3 4 5 6 7 Very much |
| I feel restless | Not at all 0 1 2 3 4 5 6 7 Very much |
| I feel self-confident | Not at all 0 1 2 3 4 5 6 7 Very much |
| Globally, I feel well | Not at all 0 1 2 3 4 5 6 7 Very much |
| I am ruminating | Not at all 0 1 2 3 4 5 6 7 Very much |
| I am able to concentrate well | Not at all 0 1 2 3 4 5 6 7 Very much |
| What am I doing (just before the beep) | Nothing;  Resting;  Eating/drinking;  Passive leisure (TV, computer, Videogames, reading);  Active leisure (walking, sports, Gardening, going out);  Travel;  Household/groceries;  Work/study;  Self-care;  Interaction/conversation;  Mobile phone/social media;  Something else |
| I can do this well | Not at all 0 1 2 3 4 5 6 7 Very much |
| I would rather do something else | Not at all 0 1 2 3 4 5 6 7 Very much |
| This activity requires effort | Not at all 0 1 2 3 4 5 6 7 Very much |
| Physically, I am active | Not at all 0 1 2 3 4 5 6 7 Very much |
| Physically, I am tired | Not at all 0 1 2 3 4 5 6 7 Very much |
| Physically, I am in pain | Not at all 0 1 2 3 4 5 6 7 Very much |
| Physically, I do not feel well | Not at all 0 1 2 3 4 5 6 7 Very much |
| Where am I? | At home;  At family or friends place;  At work or school;  Transport;  Healthcare facility;  Somewhere else indoors;  Somewhere else outdoors; |
| Who am I with? | Alone;  Partner;  Relatives living with you;  Relatives not living with you;  House/roommates;  Friends;  Classmates/colleagues;  Caregiver;  Strangers/others; |
| How many people am I with? | 1;2;3-10;over 10 |
| We are doing something together | Not at all 0 1 2 3 4 5 6 7 Very much |
| I find the people I am with pleasant | Not at all 0 1 2 3 4 5 6 7 Very much |
| I would prefer to be alone | Not at all 0 1 2 3 4 5 6 7 Very much |
| I feel connected to the people I am with | Not at all 0 1 2 3 4 5 6 7 Very much |
| I feel fine being alone | Not at all 0 1 2 3 4 5 6 7 Very much |
| I would prefer to be with others | Not at all 0 1 2 3 4 5 6 7 Very much |
| Being alone right now is my choice | Not at all 0 1 2 3 4 5 6 7 Very much |
| I feel left out | Not at all 0 1 2 3 4 5 6 7 Very much |
| I am interacting using virtual communication | Yes/No |
| I find this virtual interaction pleasant | Not at all 0 1 2 3 4 5 6 7 Very much |
| Most people would find my current situation stressful | Not at all 0 1 2 3 4 5 6 7 Very much |
| Think of the most important event that happened since the last beep. This event was: | Very unpleasant -3 -2 -1 0 1 2 3 Very pleasant |
| This event was: | Unimportant -3 -2 -1 0 1 2 3 Important |
| Since the last beep I had/taken | Food;  Caffeine;  Nicotine;  Alcohol;  Medication;  Substances;  Nothing |
| This beep disturbed me | Not at all 0 1 2 3 4 5 6 7 Very much |
| Filling out the questionnaire has influenced my mood today | Not at all 0 1 2 3 4 5 6 7 Very much |
